# From measurement to biomarker trajectories: platform-agnostic Z score analysis of serum NfL and GFAP in ocrelizumab-treated multiple sclerosis

**DOI:** 10.1007/s00415-026-13986-9

**Published:** 2026-07-10

**Authors:** Hernan Inojosa, Lars Masanneck, Katja Akgün, Ramona Hagler, Sven G. Meuth, Carolin Otto, Patrick Schindler, Karin Schulze-Bosse, Jens Kuhle, Pascal Benkert, Klemens Ruprecht, Marc Pawlitzki, Tjalf Ziemssen

**Affiliations:** 1 Center of Clinical Neuroscience, Neurological Clinic, Carl Gustav Carus University Clinic, and Centre for Tactile Internet With Human-in-the-Loop (CeTI), University of Technology, Dresden, Germany; 2 Department of Neurology and Medical Faculty, University Hospital Düsseldorf (UKD), Düsseldorf, Germany; 3 Department of Neurology, Charité-Universitätsmedizin Berlin, Corporate Member of Freie Universität Berlin Und Humboldt-Universität Zu Berlin, Berlin, Germany; 4 Central Institute of Clinical Chemistry and Laboratory Diagnostics, University Hospital Düsseldorf, Düsseldorf, Germany; 5 University Hospital Basel and University of Basel, Basel, Switzerland; 6 Department of Clinical Research, University Hospital Basel, Basel, Switzerland; 7 Division of Neuroimmunology, Department of Neurology, University of Heidelberg, Heidelberg, Germany

**Keywords:** Multiple sclerosis, Neurofilament light chain, Glial fibrillary acidic protein, Serum biomarkers, Z scores, Ocrelizumab

## Abstract

**Background:**

Serum neurofilament light chain (sNfL) and glial fibrillary acidic protein (sGFAP) are promising multiple sclerosis (MS) biomarkers, but biological confounding and inter-assay variability limit real-world use. We assessed whether Z score normalisation enables platform-agnostic monitoring in people with MS (pwMS) under ocrelizumab, and which early timepoint best stratifies subsequent biomarker trajectories.

**Methods:**

This pooled multicentre analysis of three German cohorts included pwMS treated with ocrelizumab for ≥ 12 months. Elecsys^®^ or Simoa^®^ assays were harmonised using covariate-adjusted Z scores. We evaluated biomarker 24-month trajectories and whether stratification and relative reductions predicted 24-month biomarker-status.

**Results:**

430 pwMS were included [78% relapsing MS (RMS), 22% primary progressive MS (PPMS)]. Baseline sNfL Z scores were higher in RMS; sGFAP Z scores were similar. Multivariable analyses linked higher expanded disability status scale to elevation of both biomarkers, while younger age was independently associated with higher sNfL. Following ocrelizumab, sNfL decreased in RMS while sGFAP remained stable. People with RMS and concurrent baseline elevation sustained the highest levels, diverging from the stable PPMS profile. Month-12 combined elevation best predicted persisting 24-month elevation, outperforming baseline and 6-month assessments. Crucially, failing to achieve a ≥ 50% relative reduction at month 12 strongly predicted persistent 24-month elevation for sNfL (OR 7.14, 95% CI 1.35–37.75) and sGFAP (OR 32.08, 95% CI 5.10–201.67).

**Conclusions:**

Z score normalisation enabled platform-agnostic framework for MS monitoring of downstream biological trajectories. We identify a practical 12-month exploratory responder threshold: pwMS failing to reach a ≥ 50% relative Z score reduction represent a group with persistent biomarker elevation, supporting closer clinical surveillance.

## Introduction

Multiple sclerosis (MS) is a chronic immune-mediated disease of the central nervous system (CNS) characterised by inflammatory demyelinating lesions that result in neurological damage and disability [1]. MS has a variable and unpredictable disease course, exacerbated by a lack of robust biomarkers [2]. While magnetic resonance imaging (MRI) is a cornerstone of MS diagnosis and follow-up [3, 4], frequent MRI scanning is logistically challenging and costly, and may not fully capture diffuse neurodegenerative processes or subtle progression [5]. Consequently, blood-based biomarkers that can be measured repeatedly and interpreted consistently could complement clinical and MRI assessments and enable a more patient-centred approach to MS management.

Neurofilament light chain (NfL) is a neuronal cytoskeletal protein that is released into the cerebrospinal fluid (CSF) upon neuroaxonal injury in neurological disease, such as MS, and subsequently reaches systemic circulation [6]. Serum NfL (sNfL) levels are significantly elevated in people with MS (pwMS) compared with age-matched healthy controls and have potential as a predictive marker for MS disease activity and long-term clinical outcomes [7]. Large-scale trials, such as the open-label extension of the phase III OPERA I/II and ORATORIO studies, have demonstrated that treatment with B-cell depletion with ocrelizumab significantly decreased sNfL levels, correlating with relapse activity and disease progression [8–11].

In addition, serum glial fibrillary acidic protein (sGFAP) has emerged as a promising biomarker of astrocytic activation and disability progression [12–14]. Recent evidence suggests that the combination of high sGFAP and high sNfL levels is associated with a four-fold increased risk of disability worsening [15]. This dual biomarker approach could provide deeper insights into both neuroinflammatory and neurodegenerative pathologies in MS during B-cell depletion therapy [11].

However, translating these findings into clinical reality is challenging. Although controlled trials provide high-level evidence, they typically involve strictly selected patient populations and uniform central laboratory assessments. In contrast, real-world MS populations are heterogeneous, often older, with more comorbidities and diverse treatment histories [16, 17]. Furthermore, a major barrier to real-world implementation and interpretation sNfL and sGFAP is biological and technical variability. Levels are influenced by confounders such as age, sex, body mass index (BMI), and blood volume [18, 19]. Assay heterogeneity across platforms (e.g., Simoa^®^ vs Roche Elecsys^®^) can introduce systematic differences in measured sNfL and sGFAP concentrations, such that absolute cut-offs are not comparable between laboratories and clinical centres [19–21].

To address these limitations, Z score normalisation provides a confounder-adjusted, clinically interpretable framework. By expressing biomarker values relative to age- and BMI-adjusted (and, for sGFAP, sex-adjusted) reference distributions, Z scores mitigate key confounders and improve comparability beyond absolute concentrations [22, 23].

In this multicentre real-world analysis, we applied Z score normalisation to harmonise sNfL and sGFAP measurements across diverse cohorts and assay platforms. We aimed to characterise 24-month longitudinal Z score trajectories under ocrelizumab, identify biomarker-defined subgroup patterns, and evaluate an early biomarker stratification window, including exploratory responder-based analyses, for its ability to stratify subsequent biomarker elevation—as an exploratory step toward biomarker-guided monitoring in routine care rather than a definitive predictive tool for clinical hard endpoints.

## Methods

### Study design and patient population

This is a pooled multicentre analysis of sNfL and sGFAP in pwMS in Germany who have been treated with ocrelizumab (either newly started or switched from other therapies), for at least 12 months. All patients were diagnosed with MS in accordance with McDonald 2017 criteria [24]. Treatment was administered on-label, including patients with active MS (relapsing–remitting MS [RRMS] and secondary progressive MS [SPMS] with inflammatory disease activity) as well as primary progressive MS (PPMS). Informed consent was obtained from all patients prior to study entry.

Participants were drawn from three independent cohorts: the Düsseldorf cohort, the Dresden cohort, and the CONTACT study cohort. The Düsseldorf cohort included pwRRMS enrolled in the REBELLION-MS study (NCT06586177). The CONTACT study was the first to implement Roche Elecsys^®^ blood-based assays in a real-world MS population in Germany across two centres (Düsseldorf and Berlin). The Dresden cohort represented a monocentric real-world cohort of pwMS treated with ocrelizumab. All cohorts received ethical approval from the respective ethics committees (study numbers: 2021-1475, 5951R, and 2024-2817, respectively).

PwMS from each cohort were included if essential demographic and clinical data were available for the present analysis. Required variables included: age at baseline, disease duration, MS type, sex, and expanded disability status scale (EDSS) score at baseline. For participants who switched to ocrelizumab, information on the last disease modifying therapy (DMT) was required and categorised as therapy naïve, low-efficacy DMT (interferon, glatiramer acetate, dimethyl fumarate, teriflunomide), or high-efficacy DMT (natalizumab, fingolimod, and other escalation therapies). Prior treatment with anti-CD20 antibodies or B cell-depleting therapies were not allowed.

### Clinical assessments

Clinical assessments and outcome measurements were performed by experienced neurologists certified in EDSS administration. Clinical visits including EDSS scoring were performed at baseline and every 6 to 12 months thereafter. Confirmed disability progression (CDP) was defined as an EDSS increase of ≥ 1.0 point from a baseline EDSS of ≤ 5.5, or ≥ 0.5 points if the baseline EDSS was > 5.5, confirmed at the next follow-up within 12 months.

### Biomarker sampling and quantification

Blood samples were collected before ocrelizumab treatment initiation, as well as at 6, 12, 18 and 24 months after treatment initiation, and stored locally at − 80 °C in biorepositories in Düsseldorf, Berlin and Dresden for up to 6 years. Frozen blood samples were used for retrospective analysis of sNfL and sGFAP.

sNfL and sGFAP in the Düsseldorf cohort and the CONTACT study cohort were measured at University Hospital Düsseldorf using the Elecsys^®^ NfL/GFAP immunoassays (Roche Diagnostics; research use only) [20]. In contrast, sNfL and sGFAP in the Dresden cohort were measured at the Carl Gustav Carus University Hospital Dresden using the Simoa^®^ NfL/GFAP Advantage V1 kit on the HD-X platform (Quanterix) [25].

### Statistical analysis

Normality of the data distribution was visually assessed using histograms and Quartile-Quartile plots. Based on these visual inspections, appropriate parametric or non-parametric statistical tests were applied. Formal normality testing using Shapiro–Wilk tests was not prioritized because of the large sample size and exploratory nature of the analysis. Categorical variables are summarised as counts and percentages. Continuous variables are summarised as mean with standard deviation (SD) or median with interquartile range (IQR), as appropriate. People with RRMS and active SPMS were grouped as relapsing MS (pwRMS). Missing data were not imputed; analyses used all available data for the respective timepoint/model. Given the retrospective nature of this pooled study, all statistical tests should be interpreted as exploratory.

To enable the pooling of data from the Düsseldorf, Dresden, and CONTACT cohorts, and to harmonise differences between assay platforms (Elecsys^®^ and Simoa^®^), absolute biomarker concentrations were converted into Z scores prior to analysis. sNfL and sGFAP Z scores were calculated to standardise biomarker levels relative to reference distributions while accounting for major biological covariates, including age- and BMI-related variation for sNfL and age-, BMI-, and sex-specific effects for sGFAP [20, 21]. The underlying algorithms were derived from previously published independent healthy-control reference datasets and assay-specific validation studies, rather than from the present MS cohort. Thus, Z scores express biomarker concentrations as deviations from expected values in demographically comparable healthy controls. Although Z score normalisation was used to improve comparability, this approach does not fully eliminate residual variability arising e.g. from cohort composition, pre-analytical handling or storage duration. Based on recent evidence assessed in extensive cohorts regarding predictive thresholds [23], “elevated” levels were defined using Z scores ≥ 1.5 for sNfL and ≥ 0.75 for sGFAP as cut-offs, respectively. Values below these thresholds were considered normal. Based on these cut-offs and using a scatter plot for each analysis population (pwRMS and people with PPMS [pwPPMS]), patients were stratified into four baseline quadrants for longitudinal analysis: (1) Low sNfL/Low sGFAP, (2) High sNfL/Low sGFAP, (3) Low sNfL/High sGFAP, and (4) High sNfL/High sGFAP.

Baseline differences in biomarker Z scores according to clinical factors (e.g. sex, MS type, DMT classes, age, disease duration, and EDSS) were evaluated using Student’s t-test or one-way Analysis of Variance (ANOVA), as appropriate. Tertiles were defined in the pooled cohort using the 33rd and 66th percentiles (age: 39.2 and 51.0 years; disease duration: 4.0 and 10.4 years; EDSS: 2.5 and 4.5 points). To account for multiple testing, post-hoc pairwise comparisons were adjusted using the Bonferroni correction. Pearson’s correlation coefficient and corresponding p-value were additionally calculated. Furthermore, to assess independent associations and adjust for potential confounders, multivariable linear regression analyses were performed with baseline biomarker Z scores as dependent variables, including MS phenotype, age, EDSS, disease duration, and last DMT category as covariates. For the predictive analyses, we fitted logistic regression models with 24-month biomarker elevation as the dependent variable, defined as sNfL Z score, sGFAP Z score or concurrent elevation of both biomarkers at 24 months. Separate models were run using biomarker status at baseline, month 6, and month 12 as predictors, operationalised as sNfL Z score elevation only, sGFAP Z score elevation only, or combined elevation of both biomarkers at the respective timepoint. Models were adjusted for age, baseline BMI and sex. Effect estimates are reported as odds ratios with 95% confidence intervals, and discrimination was quantified using the area under the receiver operating characteristic curve (AUC). In additional biomarker-specific responder analyses restricted to participants with elevated baseline sNfL or elevated baseline sGFAP, we evaluated whether a relative biomarker reduction from baseline to month 12 (defined as ≥ 10% or ≥ 50% reduction; responder versus non-responder) was associated with biomarker elevation at month 24. These analyses were performed using 2 × 2 contingency tables (Chi-square test or Fisher’s exact test, as appropriate) and univariable logistic regression, and we additionally derived sensitivity, specificity, positive predictive value, negative predictive value, and the Youden index from the contingency tables, and calculated AUC to assess overall discrimination. For these diagnostic analyses, the test-positive category was defined as not achieving the prespecified response threshold at month 12 (non-responder), and the outcome-positive category as biomarker elevation at month 24. The analysis of longitudinal trajectories was performed descriptively to illustrate biomarker dynamics across the stratified risk groups. To contrast the longitudinal dynamics of highly active inflammatory disease against primary progression disease, we compared the high sNfL/High sGFAP RMS subgroup (concurrent sNfL and sGFAP Z score elevation) with the overall PPMS cohort. This comparison was chosen to maximise the contrast between highly active inflammatory biology and the smouldering pathophysiology of PPMS. Due to the heterogeneity of follow-up durations and sample size limitations within biomarker quadrants, complex longitudinal trajectory models were not applied in this exploratory analysis.

Statistical analyses were performed using IBM SPSS Statistics and Python. Figures were generated using Python (Matplotlib). A two-sided p value < 0.05 was considered statistically significant.

## Results

### Study population

A total of 587 pwMS were initially considered for inclusion (Dresden n = 324, CONTACT n = 142, Düsseldorf n = 121). Due to missing data, 430 pwMS remained for the pooled analysis, comprising the Dresden cohort (n = 298), the Düsseldorf cohort (n = 96), and the CONTACT study cohort (n = 50). Of the total pooled population, 78.1% had RMS (69.1% RRMS, and 9.1% SPMS), and 21.9% had PPMS (Table 1). 29.5% of participants had previously received low-efficacy DMTs and 43% had received high-efficacy DMTs before starting treatment with ocrelizumab. During the 24-month follow-up, 19 clinical relapses and 45 CDP events were recorded.

**Table 1:** Baseline characteristics for the pooled analysis (including the Düsseldorf cohort, the Dresden cohort, and the CONTACT cohort; N = 430)

Variable	Value
Sex, n (%)	
Female	276 (64.2)
Male	154 (35.8)
MS type, n (%)	
RMS (RRMS + active SPMS)	336 (78.1)
PPMS	94 (21.9)
Number of previous DMTs, n (%)	
Naϊve	118 (27.3)
1	118 (27.6)
≥ 2	193 (45.1)
Last DMT (3-category), n (%)	
Naïve	118 (27.4)
Low-efficacy DMT	127 (29.5)
High-efficacy DMT	185 (43.0)
Last DMT (detailed), n (%)	
Naϊve	118 (27.4)
Natalizumab	51 (11.9)
Interferon	39 (9.1)
Dimethyl fumarate	44 (10.2)
Fingolimod	71 (16.5)
Glatiramer Acetate	40 (9.3)
Teriflunomide	4 (0.9)
Others (Alemtuzumab, Mitoxantrone, Infliximab, Daclizumab)	63 (14.7)
Age at baseline (years), mean (SD)	44.8 (11.9)
Disease duration (years), mean (SD)	8.6 (7.7)
EDSS at baseline, median (IQR)	3.0 (2.0–6.0)
sNfL (pg/mL), mean (SD)	11.3 (11.9)
sGFAP (pg/mL), mean (SD)	68.2 (90.6)
Z score sNfL, mean (SD)	0.58 (1.44)
Z score sGFAP, mean (SD)	0.93 (1.37)

Relapsing multiple sclerosis (RMS) includes relapsing–remitting multiple sclerosis and active secondary progressive multiple sclerosis. Low-efficacy therapies comprise interferon, glatiramer acetate, dimethyl fumarate and teriflunomide. High-efficacy therapies comprise natalizumab, fingolimod, and other escalation therapies

*DMT* disease-modifying therapy, *EDSS* expanded disability status scale, *IQR* interquartile range, *SD* standard deviation, *sGFAP* serum glial fibrillary acidic protein, *sNfL* serum neurofilament light chain, *SPMS* secondary progressive multiple sclerosis

### Baseline biomarker Z scores and clinical correlates

At baseline, pwRMS showed higher sNfL Z scores than pwPPMS (0.73 ± 1.45 vs 0.06 ± 1.29; p < 0.001), whereas sGFAP Z scores did not differ significantly between phenotypes (0.99 ± 1.40 vs 0.71 ± 1.25; p = 0.071; Table 2). Baseline sNfL and sGFAP Z scores were not associated with last DMT category (p = 0.984 and p = 0.113, respectively). Stratified analyses showed a significant age-tertile effect for sNfL Z scores (p < 0.001), as younger participants had the highest concentrations, while sGFAP did not vary by age tertiles (p = 0.154). Neither biomarker differed across disease duration tertiles (sNfL p = 0.102; sGFAP p = 0.156). Across EDSS tertiles, pwMS with higher disability levels showed higher sGFAP Z Scores (p = 0.034), whereas sNfL did not differ across these groups (p = 0.071; Table 2).

**Table 2:** Biomarkers according to baseline characteristics for the pooled analysis (including the Düsseldorf cohort, the Dresden cohort, and the CONTACT cohort; N = 430)

Group	Z score sNfL, Mean (SD)	Z score sGFAP, Mean (SD)
MS Course		
RMS (RRMS + SPMS)	0.73 (1.45)	0.99 (1.40)
PPMS	0.06 (1.29)	0.71 (1.25)
p-Value (T-Test)	< 0.001	0.071
Last DMT (3-category)		
Naϊve	0.59 (1.50)	0.85 (1.10)
Low-efficacy DMT	0.59 (1.45)	0.78 (1.46)
High-efficacy DMT	0.57 (1.40)	1.09 (1.46)
p-Value (Bonferroni), ANOVA	0.984	0.113
Age tertiles		
Lowest tertile	1.16 (1.45)^b,c^	1.06 (1.40)
Middle tertile	0.54 (1.46)^a,c^	0.99 (1.48)
Highest tertile	0.12 (1.24)^a,b^	0.76 (1.23)
p-Value (Bonferroni), ANOVA	< 0.001	0.154
Disease duration tertiles		
Lowest tertile	0.80 (1.45)	0.76 (1.22)
Middle tertile	0.51 (1.49)	0.94 (1.45)
Highest tertile	0.45 (1.38)	1.07 (1.43)
p-Value (Bonferroni), ANOVA	0.102	0.156
EDSS tertiles		
Lowest tertile	0.79 (1.51)	0.74 (1.43)^c^
Middle tertile	0.40 (1.43)	0.88 (1.25)
Highest tertile	0.56 (1.37)	1.15 (1.41)^a^
p-Value (Bonferroni), ANOVA	0.071	0.034

Stratified by tertiles of age, disease duration, and Expanded Disability Status Scale (EDSS). Tertiles were defined in the pooled cohort using the 33rd and 66th percentiles: age 39.2 and 51.0 years; disease duration 4.0 and 10.4 years; EDSS 2.5 and 4.5. Superscripts denote significant pairwise post-hoc differences (Bonferroni-corrected p < 0.05) within the corresponding tertile block only: a indicates a difference versus the lowest tertile, b versus the middle tertile, and c versus the highest tertile. Relapsing multiple sclerosis (RMS) includes relapsing–remitting multiple sclerosis (RRMS) and active secondary progressive multiple sclerosis (SPMS). Low-efficacy disease-modifying therapies comprised interferon, glatiramer acetate, dimethyl fumarate, and teriflunomide; high-efficacy therapies comprised natalizumab, fingolimod, and other escalation therapies

*DMT* disease-modifying therapy, *PPMS* primary progressive multiple sclerosis, *SD* standard deviation, *sGFAP* serum glial fibrillary acidic protein, *sNfL* serum neurofilament light chain, *SPMS* secondary progressive multiple sclerosis

In multivariable linear regression models, pwPPMS was independently associated with lower sNfL Z scores compared to pwRMS (B = − 0.53, 95% CI − 0.94 to − 0.12, p = 0.011). Younger age (B = − 0.05, 95% CI − 0.06 to − 0.03, p < 0.001) also remained a strong independent predictor. Notably, after adjusting for age and phenotype, a higher EDSS emerged as a highly significant independent predictor of elevated sNfL Z scores (B = 0.18, 95% CI 0.10–0.26, p < 0.001). For sGFAP Z scores, the multivariable model confirmed that the MS phenotype was not a significant predictor (p = 0.233). However, a higher EDSS strongly and independently predicted elevated sGFAP Z scores (B = 0.20, 95% CI 0.12 to 0.28, p < 0.001), and an independent inverse association between age and sGFAP Z scores was unmasked (B = − 0.03, 95% CI − 0.04 to − 0.02, p < 0.001). Disease duration and previous DMT category did not show significant associations with baseline Z scores in either model.

### Pooled analysis sNfL and sGFAP trajectories

**Fig. 1:**
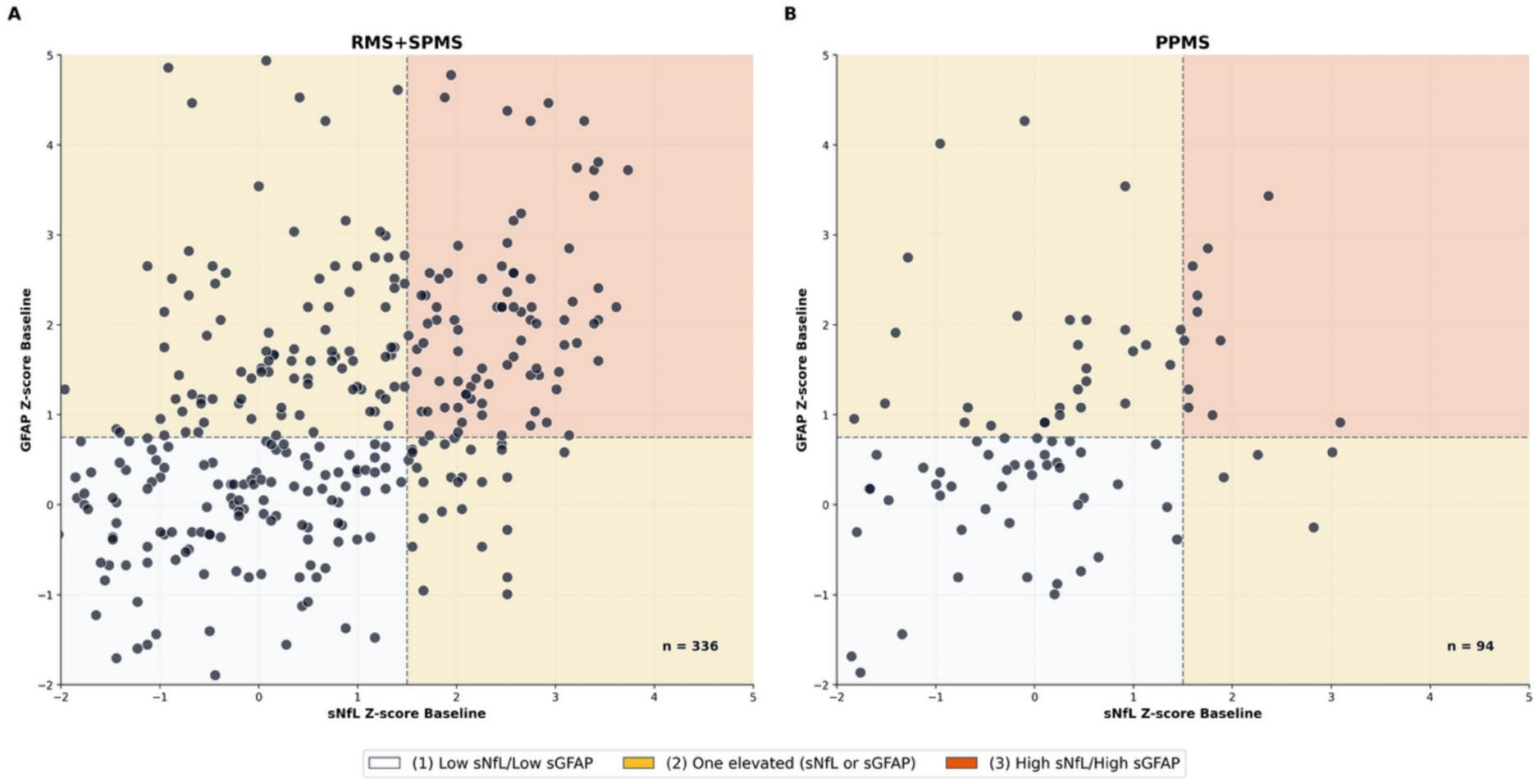
Cross-sectional distribution of sNfL and sGFAP Z scores at baseline by MS phenotype. Scatter plots of paired baseline sNfL and sGFAP Z scores in **A** people with relapsing multiple sclerosis (pwRMS; including RRMS and active SPMS) and **B** people with primary progressive multiple sclerosis (pwPPMS). Dashed lines indicate the prespecified cut-offs for biomarker elevation (sNfL Z score ≥ 1.5; sGFAP Z score ≥ 0.75), defining four biomarker quadrants. Shaded regions represent risk stratification thresholds: white indicates normal levels; light orange indicates elevation in one or both biomarkers; dark orange indicates high elevation in one or both biomarkers. Z scores are covariate-adjusted [19, 21]; a Z score of 0 reflects the expected value in the reference distribution. *sGFAP* serum glial fibrillary acidic protein, *sNfL* serum neurofilament light chain, *pwRMS* people with relapsing multiple sclerosis, *pwPPMS* people with primary progressive multiple sclerosis

**Fig. 2:**
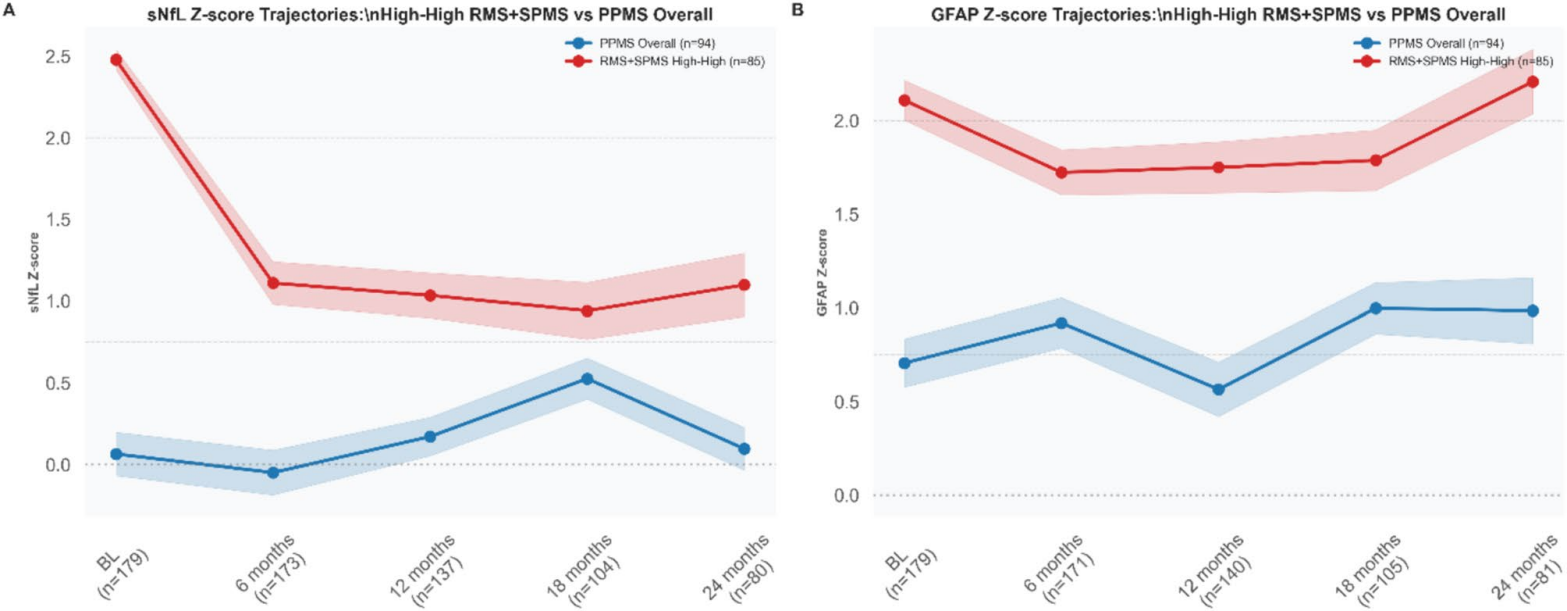
Longitudinal biomarker Z score trajectories in the RMS subgroup with concurrent baseline sNfL and sGFAP elevation compared to the overall PPMS cohort. Trajectories of **A** serum neurofilament light chain Z scores and **B** serum glial fibrillary acidic protein Z scores over 24 months following ocrelizumab initiation in the High sNfL/High sGFAP pwRMS subgroup (RRMS and active SPMS; red) compared with the overall pwPPMS cohort (blue). Panels **A**–**B** display an all-available-data approach (all available observations at each visit; sample size varies by timepoint and is shown on the x-axis). Lines represent mean Z scores and shaded bands indicate 95% confidence intervals. Trajectory plots are descriptive and were intended to illustrate longitudinal biomarker dynamics rather than formal longitudinal model-based inference. *BL* baseline, *pwPPMS* people with primary progressive multiple sclerosis, *pwRMS* people with relapsing multiple sclerosis, *sNfL* serum neurofilament light chain, *sGFAP* serum glial fibrillary acidic protein

Stratification at baseline identified four biomarker subgroups: High sNfL/High sGFAP (n = 96, 22.3%), Low sNfL/High sGFAP (n = 126, 29.3%), High sNfL/Low sGFAP (n = 30, 7.0%), and Low sNfL/Low sGFAP (n = 178, 41.4%) (Fig. 1). Longitudinal dynamics were evaluated by contrasting the subgroup with concurrent elevation (High sNfL/High sGFAP) against the pwPPMS (Fig. 2). In the High sNfL/High sGFAP RMS subgroup, sNfL Z scores decreased at 6 months and remained reduced over the 24-month period, however, they stabilised at a Z score of approximately 1, remaining clearly elevated above the healthy control average (Fig. 2A). sGFAP Z scores in this subgroup showed a transient decrease at 6 months before returning to near-baseline levels by 24 months (Fig. 2B). Among the pwPPMS cohort, biomarker trajectories remained stable over 24 months but exhibited distinct profiles: while sNfL Z scores were near 0 (corresponding to the 50th percentile of healthy controls), sGFAP Z scores were persistently elevated at approximately 1 (Fig. 2).

### Longitudinal sNfL Z score trajectories over 24 months in pwRMS by biomarker quadrants

**Fig. 3:**
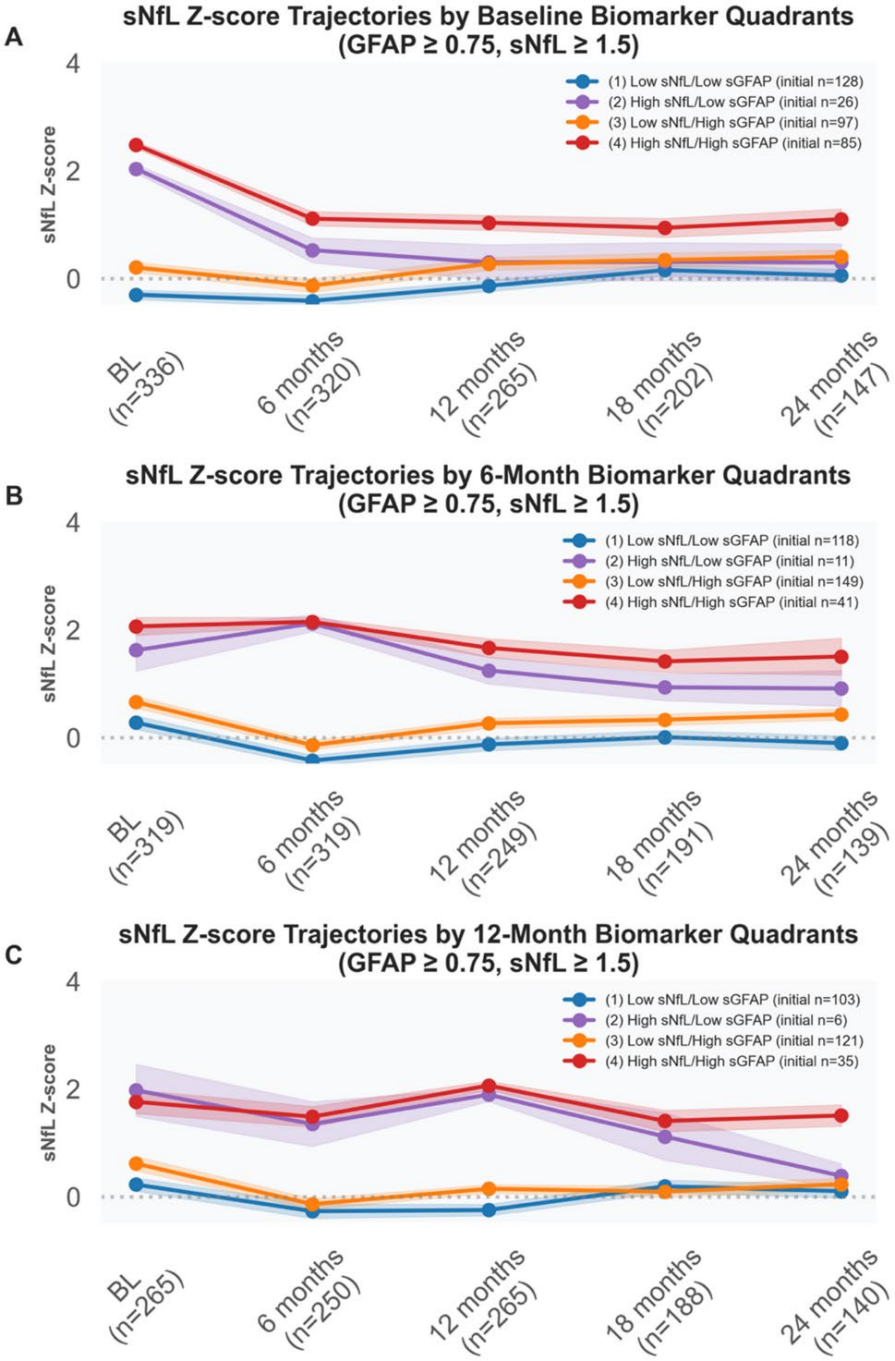
Longitudinal sNfL Z score trajectories in pwRMS stratified by biomarker status at baseline, month 6, and month 12. Evolution of sNfL Z scores over 24 months in pwRMS, stratified by combinatorial biomarker status (sNfL and sGFAP elevation) at **A** baseline, **B** month 6, and **C** month 12. Panels utilise standard elevation thresholds (sNfL Z score ≥ 1.5; sGFAP Z score ≥ 0.75). Lines represent the mean Z score for each subgroup; shaded bands indicate 95% confidence intervals. Trajectory plots are descriptive and were intended to illustrate longitudinal biomarker dynamics rather than formal longitudinal model-based inference. *sGFAP* serum glial fibrillary acidic protein, *sNfL* serum neurofilament light chain, *pwRMS* people with relapsing multiple sclerosis

Given the small pwPPMS sample size and the limited longitudinal dynamics observed, subsequent analyses were restricted to pwRMS. In pwRMS, sNfL Z scores showed a decrease at months 6, 12, and 24 in subgroups with baseline sNfL elevation (High sNfL/High sGFAP and High sNfL/Low sGFAP; Fig. 3). By contrast, subgroups without baseline sNfL elevation (Low sNfL/High sGFAP and Low sNfL/Low sGFAP) showed only minor changes in sNfL Z score over 24 months.

### Longitudinal sGFAP Z score trajectories over 24 months in pwRMS by biomarker quadrants

**Fig. 4:**
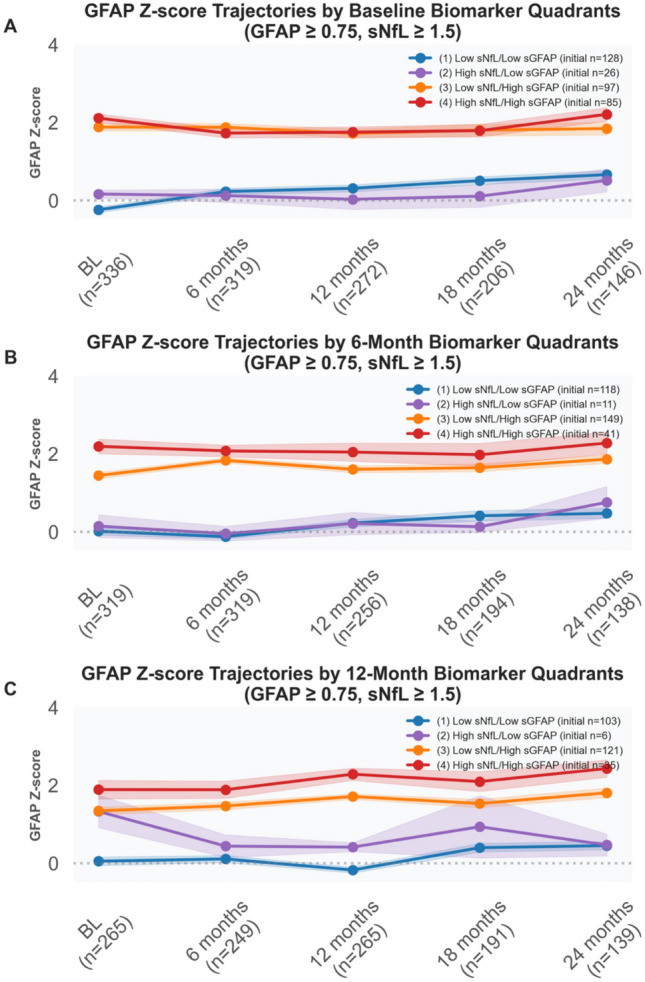
Longitudinal sGFAP Z score trajectories in pwRMS stratified by biomarker status at baseline, month 6, and month 12. Evolution of sGFAP Z scores over 24 months in pwRMS, stratified by combinatorial biomarker status at **A** baseline, **B** month 6, and **C** month 12. Similar to Fig. 3, panels utilise standard elevation thresholds. Note the relative stability of sGFAP Z scores compared to sNfL dynamics in the corresponding subgroups. Shaded bands indicate 95% confidence intervals. *sGFAP* serum glial fibrillary acidic protein, *sNfL* serum neurofilament light chain, *pwRMS* people with relapsing multiple sclerosis

sGFAP Z score trajectories remained largely stable on the group level across quadrants over 24 months (Fig. 4). Subgroups with baseline sGFAP elevation (High sNfL/High sGFAP and Low sNfL/High sGFAP) maintained elevated levels throughout follow-up (Fig. 4A and 4B), while those without baseline sGFAP elevation (Low sNfL/Low sGFAP) exhibited consistently low sGFAP Z scores over the 24-month period (Fig. 4C).

### Comprehensive predictive performance of sNfL and sGFAP

**Fig. 5:**
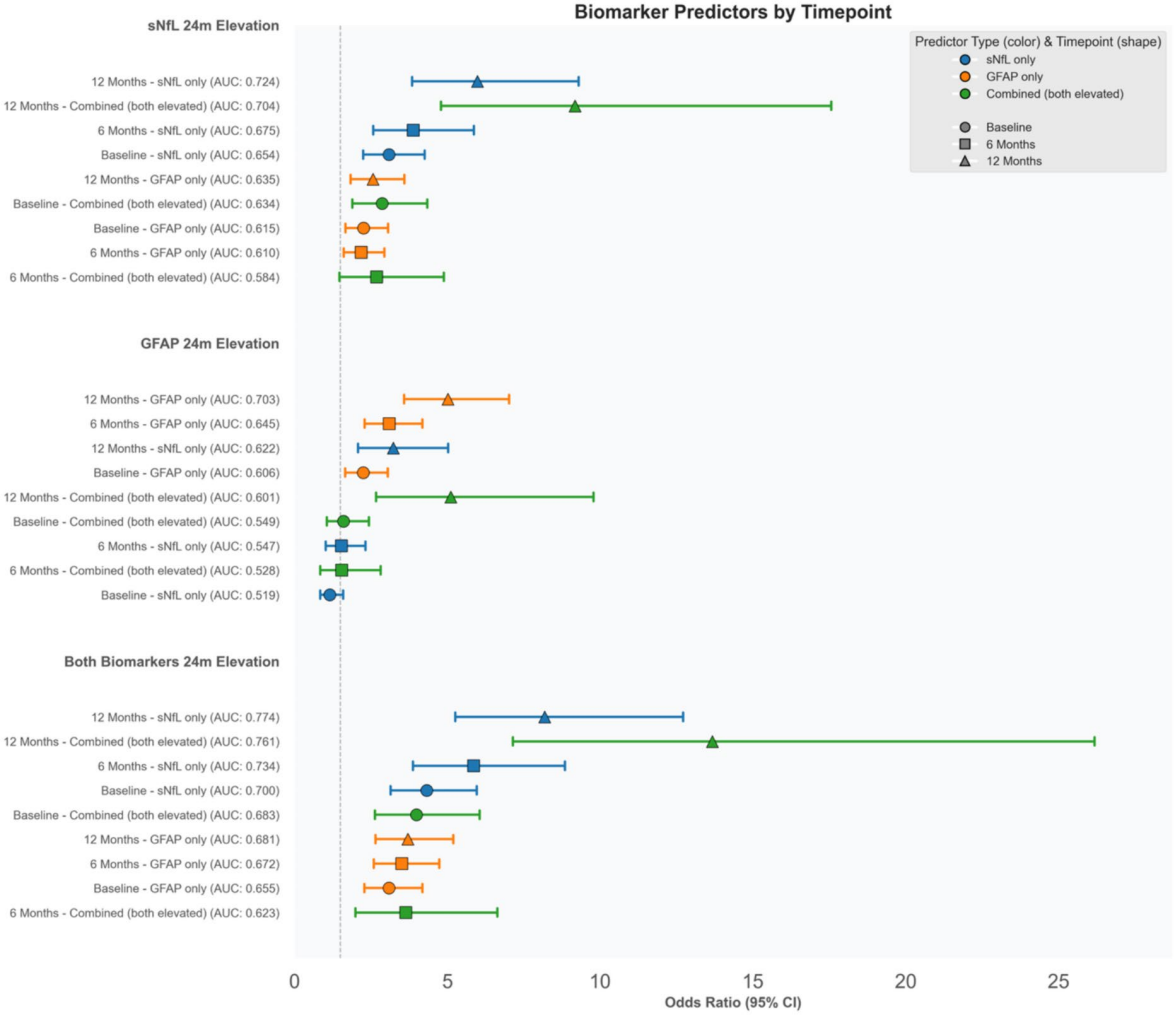
Predictive performance of early biomarker reassessment points for 24-month biomarker elevation. Logistic regression models assessing associations between earlier biomarker status and month 24 biomarker elevation. Odds ratios with 95% confidence intervals are shown for month 24 sNfL elevation, month-24 sGFAP elevation, and concurrent month-24 elevation of both biomarkers. Colours indicate predictor type (sNfL-only, sGFAP-only, combined elevation), and shapes indicate the predictor timepoint; area under the curve values are shown for each model. *AUC* area under the receiver operating characteristic curve, *CI* confidence interval, *sNfL* serum neurofilament light chain, *sGFAP* serum glial fibrillary acidic protein. Predictors are assessed at three timepoints: Baseline, Month 6, and Month 12. Error bars represent 95% confidence intervals

Figure 5 summarises logistic regression models predicting month 24 biomarker elevation using biomarker status assessed at baseline, month 6, or month 12, considering three candidate predictors at each timepoint: isolated sNfL elevation, isolated sGFAP elevation, or concurrent elevation of both. Overall, predictive performance improved with later assessments: across endpoints, the best-performing models at each timepoint increased from baseline (AUC range 0.606–0.700) to month 6 (0.645–0.734) and were highest at month 12 (0.703–0.774). Specifically, month 24 sNfL elevation was best predicted by month-12 isolated sNfL elevation (AUC 0.724), and month-24 sGFAP elevation was best predicted by month-12 isolated sGFAP elevation (AUC 0.703). For concurrent month-24 elevation of both biomarkers, discrimination was highest for month-12 isolated sNfL elevation (AUC 0.774), while month-12 concurrent elevation of both biomarkers showed the largest odds ratios but with wide confidence intervals, warranting careful interpretation. Clinically, a single biomarker check at month 12 provides the most useful exploratory signal for 24-month biological risk stratification, with sNfL informing later sNfL/dual elevation and sGFAP informing later sGFAP elevation.

### Biomarker-specific responder analyses and association with month 24 biomarker elevation

At baseline, 126 of 430 pwMS (29.3%) exhibited elevated sNfL, and 222 (51.6%) showed elevated sGFAP. Among those with elevated baseline sNfL and available month 12 data (n = 91), 42.9% failed to achieve a ≥ 50% reduction in Z score, and 13.2% failed to achieve a ≥ 10% reduction. For sGFAP (n = 168), the proportion of these non-responders was substantially higher, at 83.9% and 55.4%, respectively. In the subgroups with complete 24-month follow-up (sNfL n = 46; sGFAP n = 90), failing to reach the ≥ 50% reduction target at month 12 was strongly associated with persistent biomarker elevation at month 24 for both sNfL (OR 7.14, 95% CI 1.35–37.75) and sGFAP (OR 32.08, 95% CI 5.10–201.67) (Table 3). For both biomarkers, the 50% reduction threshold yielded high sensitivity and high negative predictive value. Applying a < 10% reduction to define treatment response improved specificity and positive predictive value but severely compromised sensitivity.

**Table 3:** Predictive performance of relative biomarker reduction at month 12 for persistent elevation at month 24

Group	Odds ratio (95% CI)	p value	Sensitivity (%)	Specificity (%)	PPV (%)	NPV (%)	Youden index
sNfL (AUC 0.784)							
< 50% reduction	7.14 (1.35–37.75)	0.021	83.3	58.8	41.7	90.9	0.42
< 10% reduction	10.33 (2.01–53.20)	0.005	50.0	91.2	66.7	80.0	0.41
sGFAP (AUC 0.828)							
< 50% reduction	32.08 (5.10–201.67)	< 0.001	92.8	71.4	97.5	45.5	0.64
< 10% reduction	10.06 (1.16–87.56)	0.036	62.7	85.7	96.5	17.1	0.48

Analyses were restricted to participants with elevated baseline levels (sNfL Z score ≥ 1.5 or sGFAP Z score ≥ 0.75) and available follow-up data at month 12 and month 24 (n = 46 for sNfL; n = 90 for sGFAP). The relative percentage reduction was calculated from baseline to month 12. For diagnostic metrics, the test-positive category was defined as failing to achieve the prespecified reduction threshold (non-responder, 10% and 50% respectively), and the outcome-positive category was defined as persistent biomarker elevation at month 24

*AUC* area under the receiver operating characteristic curve, *CI* confidence interval, *NPV* negative predictive value, *OR* odds ratio, *PPV* positive predictive value

The relative percentage reduction at month 12 showed good overall discrimination for month 24 elevation across both biomarkers (sNfL AUC 0.784; sGFAP AUC 0.828; Table 3).

## Discussion

We demonstrate that Z score normalisation overcomes inter-assay variability to enable real-world monitoring of biological trajectories and show that a 12-month “stratification window” significantly outperforms baseline assessment in predicting long-term trajectories. By pooling data across three independent cohorts and two assay platforms, we confirm that Z scores allow for clinically interpretable tracking of neuroaxonal injury and astrocytic activation in routine care.

Blood-based biomarkers such as sNfL and sGFAP can complement clinical assessment and MRI by enabling repeated, scalable monitoring of neuroaxonal injury and astrocytic activation in MS, but real-world use is hampered by biological confounding and assay variability [11]. In this pooled analysis, Z score implementation was pivotal: by normalising values to covariate-adjusted reference distributions, it enabled platform-agnostic pooling and clinically interpretable longitudinal tracking across cohorts and assays.

The present pooled analysis showed that ocrelizumab treatment was associated with a reduction in sNfL Z scores in pwRMS, likely reflecting attenuated inflammatory disease activity. These real-world findings align with clinical trial evidence, including the OPERA programme, where DMT treatment consistently lowered sNfL levels at the group level, consistent with reduced neuroaxonal injury and overall therapeutic efficacy [11, 19, 26, 27]. While these pivotal controlled trials directly linked biomarker suppression to clinical disease course, our real-world study provides a complementary, exploratory look at the temporal persistence of these biological variables themselves. Notably, this effect was observed despite substantial prior DMT exposure in our cohort—frequently including high-efficacy therapies—a context in which a pronounced biomarker shift after switching might be less expected. The observed reduction in sNfL despite this pre-treatment burden therefore supports a measurable therapeutic effect on neuroaxonal injury signals in routine care [28]. However, because our real-world registry lacks an untreated contemporary comparator arm, we cannot definitively rule out that a portion of this longitudinal decline reflects the natural history of biological fluctuations over a 24-month horizon rather than therapeutic intervention alone.

Participants with concurrent elevation of sNfL and sGFAP at baseline (High sNfL/High sGFAP) represented a distinct subgroup with the most pronounced biological burden of neuroinflammatory and astrocytic pathology. Even when sNfL declined after treatment initiation, this group remained highest across both biomarkers compared with other quadrants, stabilising however at a Z score of approximately 1. This indicates that while highly effective B-cell depletion successfully mitigates acute inflammatory waves, neuroaxonal injury remains clearly elevated above the healthy control average (Z = 0), likely reflecting ongoing axonal damage. We specifically contrasted this biologically active RMS subgroup with the PPMS cohort to highlight a key pathophysiological divergence: the sharp sNfL suppression in RMS confirms the responsiveness of acute inflammation to B-cell depletion, whereas the stable trajectory in PPMS—irrespective of treatment—points to a smouldering, compartmentally restricted pathology that is less influenced by B-cell depletion.

In pwPPMS, biomarker trajectories remained largely stable over time but revealed a clear divergence between neuroaxonal and astrocytic signals. However, this finding should be interpreted cautiously because the PPMS subgroup was relatively small (n = 94) and may have been underpowered to detect subtle longitudinal biomarker changes. While sNfL Z scores were close to 0—corresponding to the 50th percentile of matched healthy controls—sGFAP Z scores were persistently elevated at approximately 1. This divergent profile reflects the clinical pathophysiology of primary progressive disease: the relative absence of acute, B-cell-mediated inflammatory waves keeps sNfL within the normal physiological range, whereas the compartmentally restricted, smouldering astrogliosis driving continuous clinical progression is captured by the elevated sGFAP. This stability underscores that the acute inflammatory component driving the rapid sNfL suppression in the High sNfL/High sGFAP RMS subgroup plays a subordinate role in the primary progressive phenotype, possibly correlating with milder inflammatory components [6, 11, 29]. Our data also highlight that younger age is an independent driver of sNfL elevation, whereas sGFAP elevation is more closely tied to disability accumulation across both phenotypes.

Our analysis supports an integrative interpretation of the biological roles of each biomarker. Although sNfL Z scores were highest in the youngest patients in univariable comparisons, reflecting the intense inflammatory activity characteristic of early RMS, both biomarkers were independently associated with EDSS after adjustment for age and phenotype. Furthermore, adjusting for EDSS unmasked an independent inverse association between age and sGFAP, suggesting that the raw age effects are heavily confounded by disease progression. Together, these findings suggest that sGFAP may be more closely related to disability-associated astrocytic activation, whereas sNfL may capture both inflammatory neuroaxonal injury and disability-related tissue damage, support combined interpretation of both biomarkers [6, 11, 30]. Crucially, the persistent independent effect of age within our multivariable models indicates that Z score calculation may improve cohort comparability, but does not entirely eliminate biological age-dependent variability, which likely reflects residual immunosenescence and ongoing subclinical neurodegeneration.

Prior work supports this complementary concept: while sNfL is more closely linked to acute inflammatory activity and relapse risk, higher sGFAP has been associated with CDP and future progression, and its longitudinal behaviour may vary by DMT class—for example, a fingolimod-treated cohort showed sGFAP Z scores predicting disability progression whereas sNfL predicted relapses, alongside a modest treatment-associated decline in sGFAP that may not be observed with other DMTs, including ocrelizumab [23]. In this context, persistently elevated sGFAP together with absent sNfL suppression after DMT initiation has been linked to higher progression risk.

Biomarker status at month 12, particularly combined elevation of sNfL and sGFAP, showed the strongest association with subsequent month 24 biomarker elevation and the best overall discrimination compared with baseline or month 6. This supports month 12 as a pragmatic reassessment point to capture treatment response and ongoing disease biology in real-world care. Integrating sNfL and sGFAP assessment alongside MRI could therefore enable more continuous, scalable monitoring of neuroaxonal injury and astrocytic activation, complementing imaging follow-up and refining risk stratification for activity and progression [7]. Clinically, a decline in sNfL Z score after DMT initiation may provide an exploratory signal of reduced neuroaxonal injury, whereas persistently elevated sNfL at 12 months may identify individuals with ongoing biomarker elevation who warrant closer clinical and paraclinical evaluation in future validation studies [11]. Biologically, a persistent elevation of these markers at 12 months identifies a subgroup with sustained biological activity, though further validation is strictly required before these cut-offs can be utilised to guide actual therapeutic switches or routine clinical decisions.

A key practical barrier to implementing blood biomarkers in routine care is variability across assay platforms and laboratories. In this context, both Elecsys^®^ and Simoa^®^ offer clinically attractive characteristics, including user-friendly workflows, short turnaround times, and high analytical sensitivity and precision, and their results show good inter-assay correlation [30]. However, absolute (raw) sNfL and sGFAP concentrations remain difficult to interpret across settings because they do not account for major biological covariates such as age, sex, and BMI and lack harmonised cut-offs across platforms. Moreover, raw sNfL values have been shown to provide less accurate estimation and prognostication of ongoing neuroaxonal injury than covariate-adjusted Z scores [22].

In our study, Z score normalisation enabled platform-agnostic, cross-cohort comparisons and supported standardised, clinically interpretable longitudinal monitoring of sNfL and sGFAP [21]. This facilitates translation beyond research settings by providing a pragmatic framework for routine biomarker-based monitoring of disease course, treatment response, and patient risk. However, although Z score normalisation enabled a common analytical scale by accounting for major biological covariates and platform-specific reference distributions, it cannot fully exclude residual bias from cohort composition, pre-analytical procedures, storage conditions, or assay-specific effects.

Certain limitations of this retrospective pooled analysis should be acknowledged. First, potential selection bias cannot be excluded due to missing data and the requirement for available longitudinal samples. Second, we did not assess the predictive value of Z scores for disease activity and CDP in this analysis. Given the high efficacy of ocrelizumab in suppressing inflammatory activity and the relatively short follow-up of 24 months, CDP events were infrequent, limiting the statistical power for meaningful clinical prediction models. As our cohort experienced low relapse and progression rates, our models demonstrate the persistence of biological trajectories rather than clinical progression. Similarly, the follow-up duration may be insufficient to capture slower longitudinal biomarker changes, particularly for sGFAP and progression-related biology. Furthermore, our responder analyses evaluating relative Z score reductions at month 12 were based on highly restricted sample size due to strict biobanking availability requirements, resulting in relatively large odds ratios and threshold-based performance metrics statistically unstable and exploratory. Finally, MRI outcomes were not analysed. Because MRI acquisitions in standard routine registries occur at irregular intervals on heterogeneous scanner types (1.5 T vs 3 T) without standardised central protocols, longitudinal imaging correlation was not feasible, leaving the complete neuroimaging validation of these biological trajectories outside the scope of the current work. However, our primary objective was not to reproduce MRI-based endpoints, but to characterise biomarker dynamics and demonstrate cross-cohort comparability across assay platforms. This approach mirrors the requirements of routine care, where MRI is often obtained at irregular settings or intervals, highlighting the need for scalable, standardised blood-based monitoring. Consequently, future studies with longer observation periods and rigorous structural imaging integration are necessary to validate sNfL and sGFAP Z scores against clinical disability outcomes in treated populations.

In conclusion, by pooling three independent cohorts (Düsseldorf, Dresden, CONTACT) and incorporating both Elecsys^®^ and Simoa^®^ assays, we demonstrate that Z score normalisation enables robust, platform-agnostic interpretation of sNfL and sGFAP in routine care. These findings support sNfL and sGFAP Z scores as scalable tools to complement established monitoring and to refine individual risk stratification and treatment evaluation in MS.

## Data Availability

The original contributions presented in this study are included in the article. The data are not publicly available due to restrictions regarding data protection, as they include information that could compromise the privacy of research participants. Anonymised data supporting the findings of this study are available on request from the corresponding author.
